# The Bioactive Secondary Metabolites from *Talaromyces* species

**DOI:** 10.1007/s13659-015-0081-3

**Published:** 2016-01-08

**Authors:** Ming-Ming Zhai, Jie Li, Chun-Xiao Jiang, Yan-Ping Shi, Duo-Long Di, Phillip Crews, Quan-Xiang Wu

**Affiliations:** State Key Laboratory of Applied Organic Chemistry, College of Chemistry and Chemical Engineering, Lanzhou University, Lanzhou, 730000 People’s Republic of China; Key Laboratory of Chemistry of Northwestern Plant Resources and Key Laboratory for Natural Medicine of Gansu Province, Lanzhou Institute of Chemical Physics, Chinese Academy of Sciences, Lanzhou, 730000 People’s Republic of China; Department of Chemistry and Biochemistry, University of California Santa Cruz, Santa Cruz, CA 95064 USA

**Keywords:** *Talaromyces*, Secondary metabolites, Biological activities

## Abstract

**Abstract:**

The focus of this review is placed on the chemical structures from the species of the genus *Talaromyces* reported with reference to their biological activities. 221 secondary metabolites, including 43 alkaloids and peptides, 88 esters, 31 polyketides, 19 quinones, 15 steroid and terpenoids, and 25 other structure type compounds, have been included, and 66 references are cited.

**Graphical Abstract:**

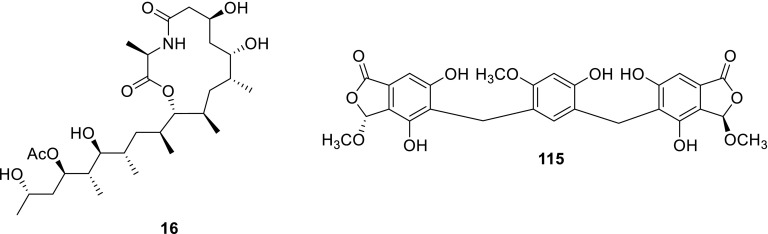

## Introduction

The name *Talaromyces* is derived from the Greek word for ‘basket’, which aptly describes the body in which ascospores are formed. In the past, species producing sexual stages with *Penicillium* anamorphs have been classified in *Eupenicillium* and *Talaromyces*. After July 2011, species formally classified in the *Penicillium* subgenus *Biverticillium* were classified in *Talaromyces*. The situation is complicated by the fact that many species now classified in *Talaromyces* will continue to be sought as *Penicillium* species in identifications [1]. So in this review, all of the papers which reported the secondary metabolites from the species named *Talaromyces* were covered.

The genus *Talaromyces* (Trichocomaceae) is an important fungal genus because of its ubiquity which were isolated from soil, plants, sponges, and foods. Some of the species are heat resistant. Some of the species are famous because of their enzymes applicable in the synthesis of saccharides, preparation of chiral building blocks or biotransformations, and for its application in pest biocontrol. Many of its species are used in food and agricultural production. Interestingly, the *T. pinophilus* strain EMOO 13–3 is able to degrade agricultural waste [2]. However, although endemic in maize, *T. funiculosus* also occurs in a wide range of other foods and sometimes causes spoilage [1]. Considering their importance, members of this genus have attracted the attention of chemists. Many studies have focused on the secondary metabolites.

## The Secondary Metabolites

The secondary metabolites of *Talaromyces* mainly include alkaloids, peptides, lactones, polyketides, and miscellaneous structure type compounds. *T. flavus*, a microorganism remarkable for its secondary metabolites with unique biological activities, is the commonest species of the genus *Talaromyces* [3]. All of the natural products from the species of this genus are classified. The reported bioactivities are also represented below.

### Alkaloids and Peptides

Alkaloid is a kind of important natural products. Many alkaloids have various kinds of biological activities, such as antibacterial, antifungal, cytotoxic, and nematicidal. The structures of alkaloids isolated from *Talaromyces* species are mainly nitrogen heterocyclic derivatives.

Two prenylated indole alkaloids, talathermophilins A and B (**1** and **2**), were isolated from a thermophilic fungus *T. thermophilus* strain YM1-3. And the ratio of **1** and **2** in the culture broths was unexpectedly rather constant (about 2:3), which even remained unchanged despite the addition of exogenous **1** or **2** suggesting that talathermophilins might be of special function for the extremophilic fungus. Those both compounds showed nematicidal toxicity (ca. 38 and 44 % inhibition, respectively) toward the worms of the free-living nematode *Panagrellus redivivus* at a concentration of 400 μg/mL for 72 h. The family of prenylated indole alkaloids is a well-known group of secondary metabolites mainly produced by *Aspergillus* and *Penicillium* sp. This is a first report about pyranoindol alkaloids from *Talaromyces* [4]. Other fourindole alkaloids with various levels of prenylation, talathermophilins C–E (**3**–**5**) and cyclo (glycyltryptophyl) (**6**), from the thermophilic fungus *T. thermophilus* strain YM3-4 which was collected in hot springs, were also elucidated by the same research group in 2011 [5]. Interestingly, authors found that only a very small group of amino acids (glycine, alanine, proline, and its derivatives) could be naturally chosen as a starting building block to form the 2,5-diketopiperazine with tryptophan [4, 5].
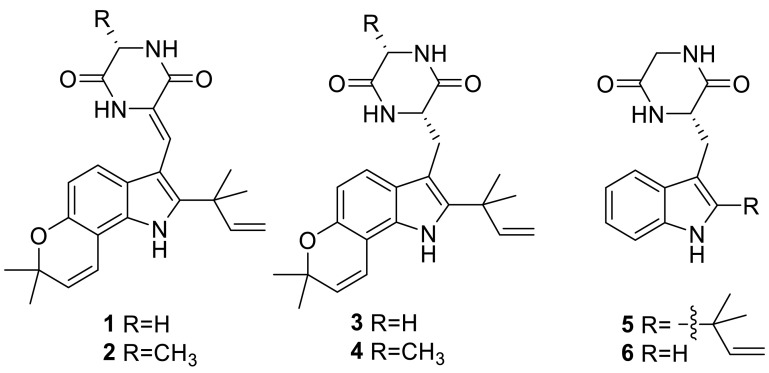


Seven known indole alkaloids (**7**–**12**) were obtained from the culture of the alga-endophytic fungus *Talaromyces* sp. cf-16. Bioassay results showed that **9** was more toxic to brine shrimp than the other compounds, and **8**, **9**, and **10** could inhibit *Staphylococcus aureus* [6].
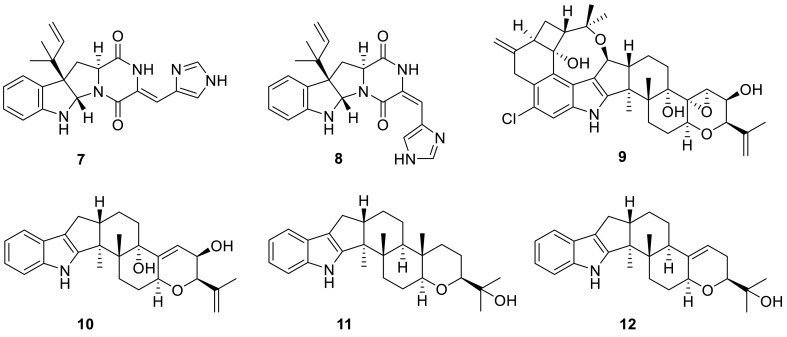


Three known diketopiperazines, cyclo(l-proline-l-leucine) (**13**), cyclo(l-proline-l-phenylalanine) (**14**), and cyclo(l-tyrosine-l-phenylalanine) (**15**), were isolated from the methanolic extracts of the green Chinese onion-derived fungus *T. pinophilus* AF-02 [7].
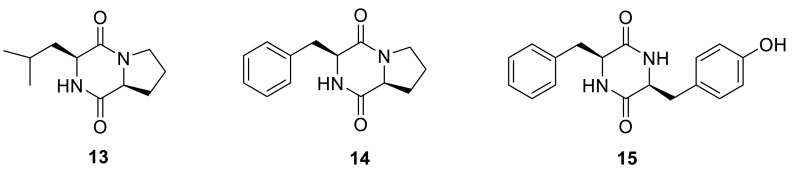


An unprecedented class of PKS-NRPS hybrid metabolites possessing a 13-membered lactam-bearing macrolactone, thermolides A–F (**16**–**21**), were also obtained from *T. thermophilus* YM3-4. They showed that compounds **16** and **17** displayed potent inhibitory activity against three notorious nematodes with LC_50_ values of 0.5–1 μg/mL, as active as commercial avermectins. This is the first report on the discovery of hybrid macrolides from a fungus origin [8]. Afterwards, a combination of chemical screening, genome analyses, and genetic manipulation led to the identification of the thermolide biosynthetic genes from sister thermophilic fungi *T. thermophilus* and *Thermomyces lanuginosus* C5. And a novel macrolactone, thermolide G (**22**), was obtained from the cultural broth of *Thermomyces lanuginosus* C5. Their results revealed the first fungal hybrid iterative polyketide synthase–nonribosomal peptide synthetase (PKS–NRPS) genes involved in the biosynthesis of bacterial-like hybrid macrolactones instead of typical fungal tetramic acids-containing metabolites [9].
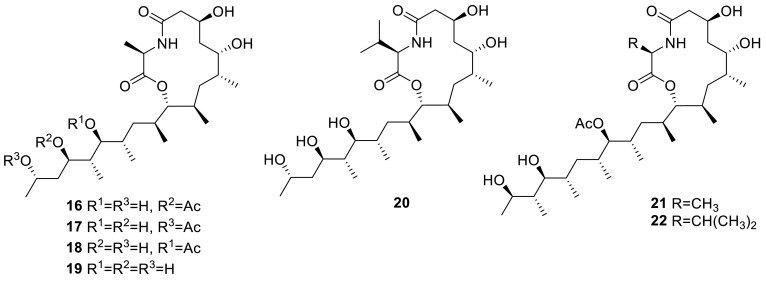


Four new tetramic acid derivatives, talaroconvolutins A–D (**23**–**26**), along with a known mitorubrin derivative, ZG-1494R (**27**), were isolated from the strain *T. convolutes* by the group of Shun-ichi Udagawa in 2000. The antifungal activity of the talaroconvolutins against the pathogenic fungi *Aspergillus fumigates*, *A. niger*, *Cryptococcus albicans*, and *C. neoformans*, was determined. And the results showed that talaroconvolutins B (**24**) and C (**25**) and ZG-1494R (**27**) inhibited the growth of *A. fumigatus*, *A. niger*, and *C. albicans* [10].
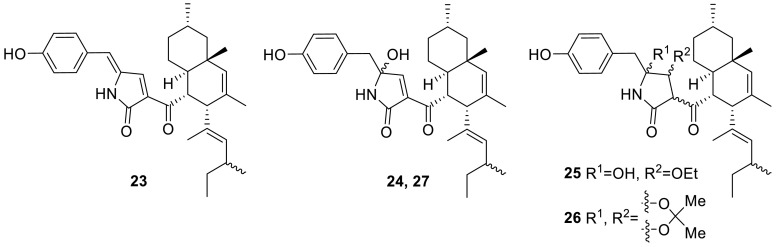


Four new drimane sesquiterpene lactones conjugated with *N*-acetyl-l-valine, minioluteumides A–D (**28**–**31**), and three known compounds, purpuride (**32**), berkedrimane B (**33**), and purpuride B (**34**), were isolated from the marine fungus, *T. minioluteus* (*P. minioluteum*) by the group of Prasat Kittakoop. The structure **28** was elucidated by single crystal X-ray analysis. **28**, **31** and **33** showed cytotoxic activity against HepG2 with IC_50_ ranges of 50.6–193.3 μM, but **28**–**34** did not shown any inhibit activity to caspase-3 [11].
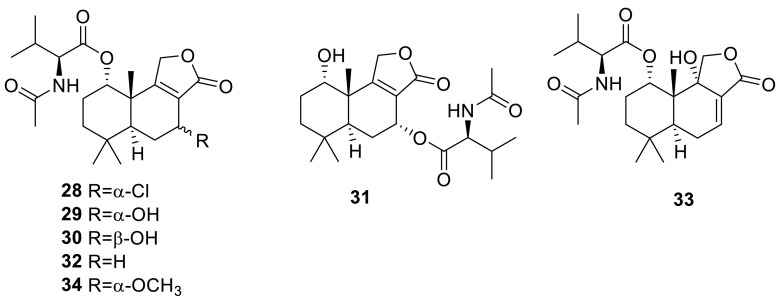


A peptide analogue *N*-benzoylphenylalanyl-*N*-benzoylphenylalaninate (**35**) was isolated from the fungus *T. thailandiasis*, which was firstly found from a higher plant, *Croton hieronymi* [12]. Two new cyclic peptides, talaromins A and B (**36** and **37**) were yielded from the endophytic fungus *T. wortmannii*, isolated from *Aloe vera* by the group of Peter Proksch and Abdessamad Debbab. Both cyclopeptides contain ring systems comprised of six α-amino acid residues connected to β-amino acid. The absolute configurations of the α-amino acids were determined by Marfey’s method. Both compounds showed no activity when evaluated for their cytotoxicity against L5178Y mouse lymphoma cells and no antibacterial activity against a broad spectrum of bacterial strains up to a concentration of 64 μg/mL [13]. 9-(3-L-alanylamino-3-carboxypropyl)adenine (NK374200, **38**) with a peptidyl adenine nucleus was isolated from the culture broth of the fungus *Talaromyces* sp., which had been isolated from a soil sample. **38** was screened in various biological assay systems, and found to have anti-mosquito larval activity [14].
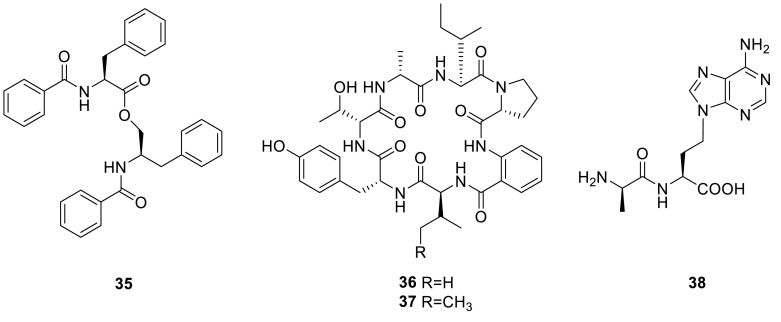


Two quinazoline alkaloids, 2-[(*S*)-hydroxy(phenyl)methyl]-3-methylquinazolin-4(3*H*)-one (**39**) and 2-[(*R*)-hydroxy(phenyl)methyl]-3-methylquinazolin-4(3*H*)-one (**40**), and a pyridone derivative (**41**), were isolated and identified in a culture of the alga-endophytic fungus *Talaromyces* sp. cf-16 for the first time. Following chiral column chromatography, compounds **39** and **40** were identified as enantiomers by spectroscopic analyses and quantum chemical calculations [6].
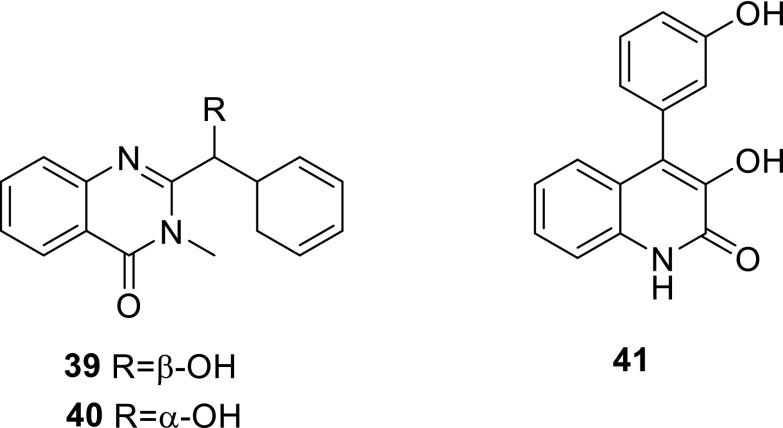


(*E*)-3-(2,5-dioxo-3-(propan-2-ylidene)pyrrolidin-1-yl)acrylic acid (**42**) was isolated from the ethyl acetate extract of the culture broth of *T. verruculosus*, a rhizosphere fungus of *Stellera chamaejasme* L.In the antimicrobial activities, **42** gave slight active against the plant pathogenic fungi, *Alternaria solani*, *Valsa mali*, *Curvularia lunata*, and *Botryosphaeria berengeriana*, at 100 μg/mL and its MIC values against pathogenic bacteria, *Straphylococcus aureus* and *Escherichia coli*, were more than 100 μg/mL [15]. Emerin (**43**) was obtained from the extract of *T. flavus* IFM52668, and showed no activity against pathogenic filamentous fungi, *Aspergillus fumigatus* and *A. niger*, and pathogenic yeasts, *Candida albicans* and *Cryptococcus neoformans*, at 200 μg/disc [16].
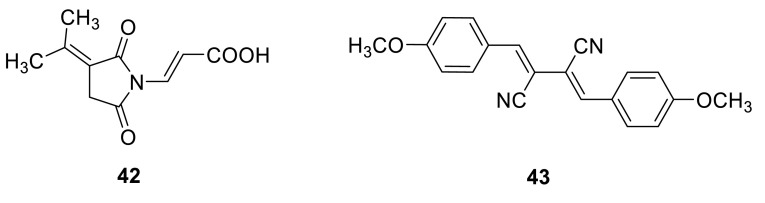


### Esters

The secondary metabolites of *Talaromyces* are mainly esters, including macrolides, linear polyesters, aromatic lactones, coumarins, phthalides, and five/six-membered saturated lactones.

Four novel 22-membered triene macrolides, wortmannilactones A–D (**44**–**47**), were obtained from the fungus *T. wortmannii* which isolated from a soil sample collected in China’s Yunnan province. **44**–**47** were screened for cytotoxic activity against a panel of human cancer cell lines (HCT-5, HCT-115, A549, MDA-MB-231, and K562). The IC_50_ values range from 28.7 to 130.5 μM [17]. Vermiculine (**48**), a 16-membered macrolide dilactone antibiotic had been found in crystalline solid from *T. wortmannii*, isolated from a soil sample [18].

Seven 15G256 macrolidepolyesters, 15G256ι (**49**), 15G256β (**50**), 15G256α (**51**), talapolyester E (**52**), 15G256α-1 (**53**), talapolyester F (**54**), and 15G256ω (**55**), were isolated from the wetland soil-derived fungus *T. flavus* BYD07-13 by Chinese researchers. Among these compounds, **50** and **55** exhibited significant activity against MCF-7 cell line with the IC_50_ of 3.27 and 4.32 μM, respectively [19]. **51** [20, 21] and **53** [22] were also isolated from the soil-derived fungus *T. flavus* FKI-0076 by Japanese researchers. In the course of screening for synergist effects with clinic-used miconazole as well as antifungal agent, **51** was showed that can inhibit *Bacillus subtilis* (IC_50_ 15 mg/L), *Staphyloccus aureus* (IC_50_ 90 mg/L), *Micrococcus luteus* (IC_50_ 100 mg/L), *Mucorracemosus* (IC_50_ 40 mg/L) [20]. As proposed by Schlingmann, 15G256 polyesters are biosynthetically assembled by alternately linking 2,4-dihydroxy-6-(2-hydroxypropyl)benzoic acid and 3-hydroxybutyric acid moieties [23].
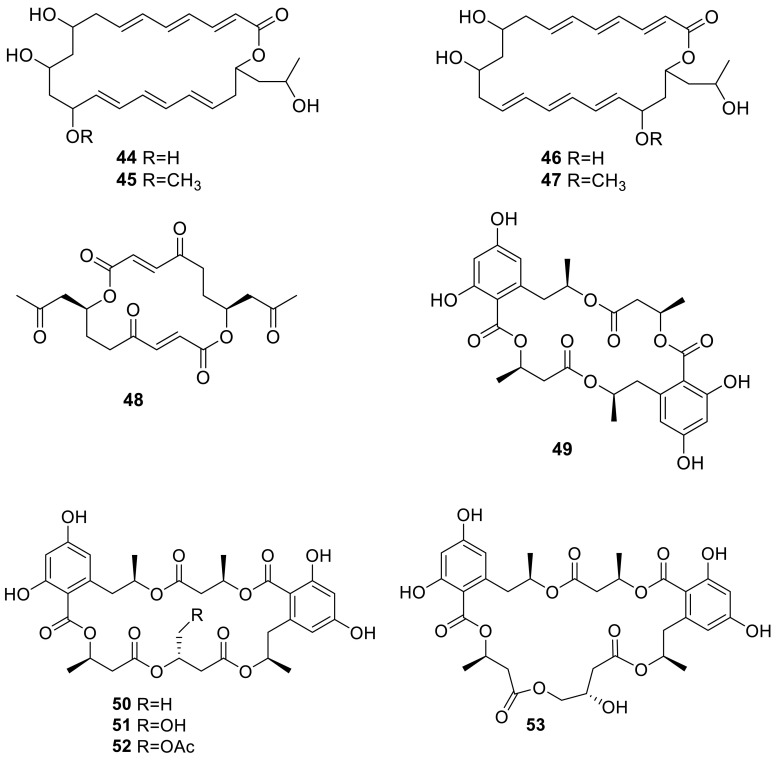

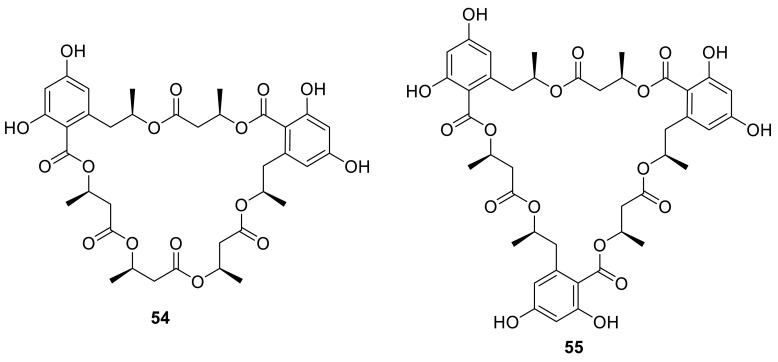


Four new linear polyesters, talapolyesters A–D (**56**–**59**), together with six known compounds (**60**–**65**), were isolated from the wetland soil-derived fungus *T. flavus* BYD07-13. Those compounds contained both 2,4-dihydroxy-6-(2-hydroxypropyl)benzoic acid or its derivatives and 3-hydroxybutyric acid or its derivatives. The cytotoxicity against five tumor cell lines of those compounds was examined, but all polyesters were inactive (IC_50_ > 40 μM) as compared to cisplatin [19].
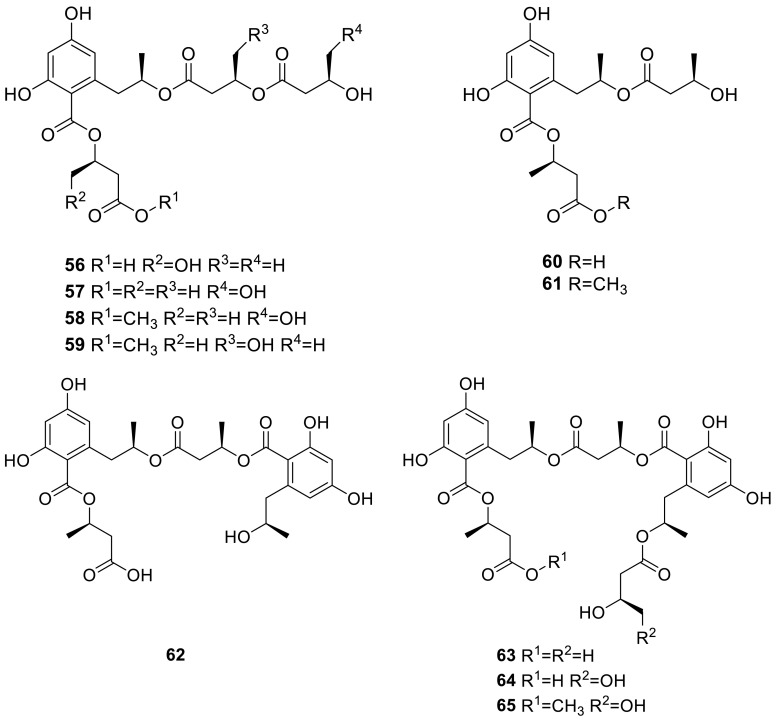


Three new oxaphenalenone dimers, bacillosporins A–C (**66**–**68**), were isolated from *T. bacillosporus* NHL 2660. **66** had the antibacterial activity against *Bacillus subtilis* and *Sarcina lutea* [24]. Other oligophenalenone dimers, bacillisporins D and E (**69** and **70**) and duclauxin (**71**), were isolated from the fungus *T. bacillisporus* from a soil sample. They were screened for in vitro cytotoxicity again three human tumor cell lines MCF-7, NCI-H-460 and SF-268, and **71** exhibited moderate inhibitory effects against all three cell lines but **70** showed little activity [25]. In 2015, two new oxaphenalenone dimers, talaromycesone A (**72**) and talaromycesone B (**73**), were isolated from the culture broth and mycelia of a marine fungus *Talaromyces* sp. strain LF458. **72** exhibited potent antibacterial activities with IC_50_ 3.70 μM against human pathogenic *Staphylococcus* strains, and **72** also displayed potent acetylcholinesterase inhibitory activities with IC_50_ 7.49 μM [26].
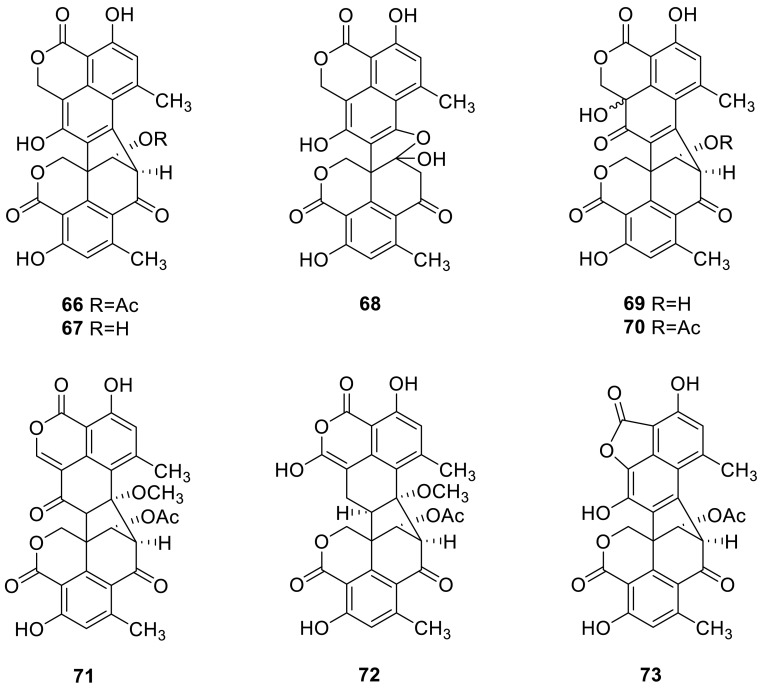


Antibacterial binaphtho-α-pyrones, talaroderxines A and B (**74** and **75**) were isolated from a new heterothallic ascomycetous fungus, *T. derxii*, cultivated on rice. The antibacterial activities of the metabolites from *T. derxii* and their derivations against *Bacillus subtilis* indicated that only talaroderxine, the mixture of **74** and **75**, showed antibacterial activity, which was almost as strong as that of viriditoxin. And talaroderxine had inhibitory activity toward 5-lipoxygenase, its IC_50_ value was determined as 3.8 × 10^−6^ M [27].

Eight new dinapinones, AB1, AB2, AC1, AC2, AD1, AD2, AE1 and AE2 (**76**–**83**) were obtained from the culture broth of *T. pinophilus* FKI-3864. All these dinapinones possessed the same biaryl dihydronaphthopyranone skeleton consisting of a heterodimer with one monapinone A and one different monapinone. The effect of dinapinones was evaluated on the synthesis of [^14^C] triacylglycerol (TG) and [^14^C] cholesterol ester from [^14^C] oleic acid in CHO-K1 cells and the results indicated that dinapinone (**77**) showed potent inhibition of TG synthesis in intact mammalian cells with an IC_50_ value of 1.17 μM, whereas the other dinapinones showed weak inhibition of TG synthesis [28].
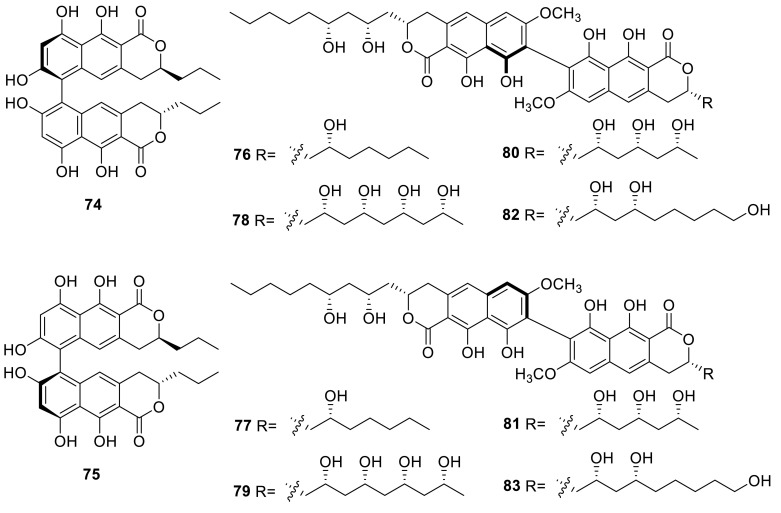


Sixdiphenyl ether lactone derivatives (**84, 85** and **86**–**88**) and AS-186c (**89**) were isolated from amarine fungus *Talaromyces* sp. strain LF458. **89** exhibited potent antibacterial activities with IC_50_ 1.34 μM against human pathogenic *Staphylococcus* strains, potent acetylcholinesterase inhibitory activities with IC_50_2.60 μM, and phosphodiesterase PDE-4B2 inhibitory activities with IC_50_2.63 μM [26]. Penicillide and dehydroisopenicillide (**84** and **85**) were isolated from *T. derxii* cultivated on rice [29]. Penicillide was also isolated from the methanolic extracts of the green Chinese onion-derived fungus *T. pinophilus* AF-02 [7].
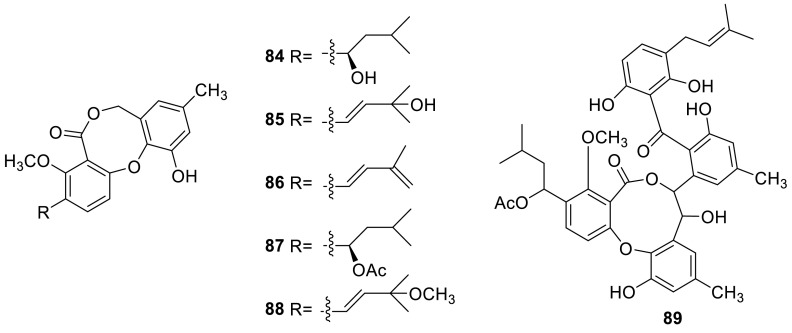


A coumarin **90** was obtained from the organic extracts of the soil fungus *T. flavus* [30]. Two new coumarins, talacoumarins A (**91**) and B (**92**), were isolated from the ethyl acetate extract of the wetland soil-derived fungus *T. flavus* BYD07-13. They were evaluated for anti-Aβ42 aggregation, cytotoxic, and antimicrobial activities and the results showed that **91** and **92** had moderate anti-Aβ42 aggregation activity, and this was the first report on the Aβ42 inhibitory aggregation activity of coumarins [31].
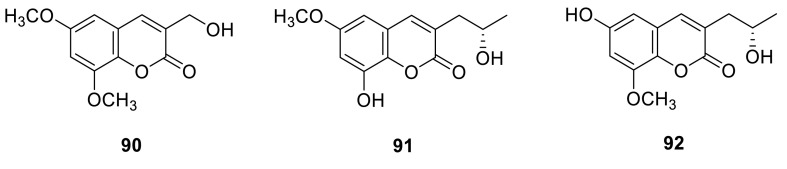


An *O*-methylated 3,4-dihydroisocoumarin **93** was isolated from a previously undescribed fungus *T. thailandiasis* [12]. An isocoumarin derivate (**94**) was isolated from the ethyl acetate extract of the culture broth of *T. verruculosus*, a rhizosphere fungus of *Stellera chamaejasme* L. **94** exhibited the significant activities in vitro against *Staphylococcus aureus* and *Escherichia coli*, with MIC values of 2.5 and 5.0 μg/mL, respectively. And for the plant pathogenic fungi, **94** disclosed significant growth inhibitions of 92.6 ± 2.1, 97.3 ± 3.3, 87.2 ± 2.8 and 94.9 ± 1.9 % at 50 μg/mL against *Alternaria solani*, *Valsa mali*, *Curvularia lunata* and *Botryosphaeria berengeriana*, respectively [15]. Two isocoumarin derivates (**95** and **96**) were isolated from the organic extracts of the soil fungus *T. flavus* [30]. Sclerotinin A (**97**) and alternariol (**98**) were isolated from the methanolic extracts of the green Chinese onion-derived fungus *T. pinophilus* AF-02 [7].

Merodrimanes, thailandolides A (**99**) and B (**100**), a drimane linked through a tertiary oxygen to the dihydroisocoumarin, were isolated from a previously undescribed fungus *T. thailandiasis* [12]. A new meroterpenoid, chrodrimanin C (**101**) together with chrodrimanins A and B (**102** and **103**) from the strain YO-2 of *Talaromyces* sp. Chrodrimanin B exhibited insecticidal activity with an LD_50_ value of 10 μg/g of diet, while chrodrimanins A and C were inactive [32]. Four new meroterpenoids, named chrodrimanin D–G (**104**–**107**), and a known compound chrodrimanin H **(108)** were also isolated from the strain YO-2 of *Talaromyces* sp. Chrodrimanins D, E and F (**104**–**106**) showed insecticidal activity against silkworms with respective LD_50_ values of 20, 10 and 50 μg/g of diet [33].
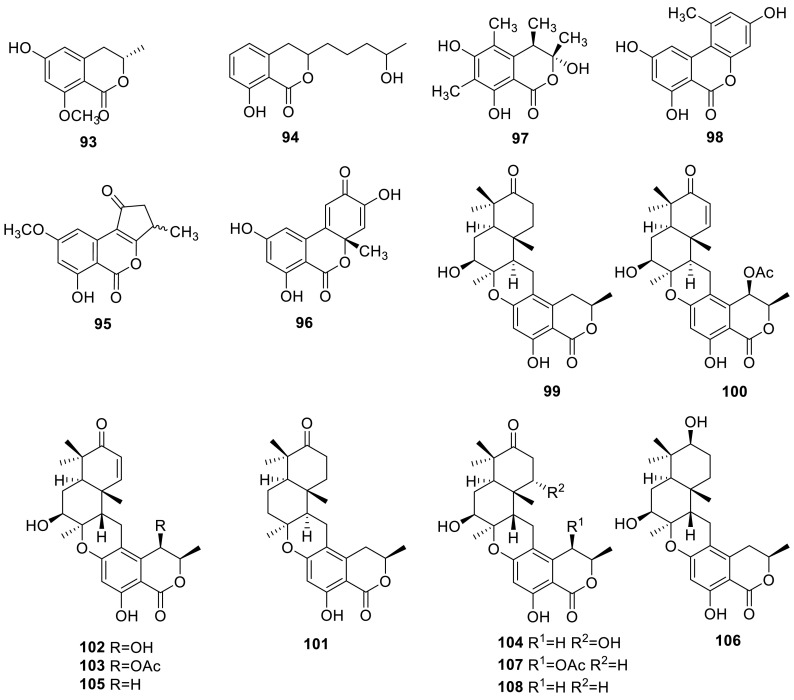


A phthalide derivative **109** and a spiro-phthalide derivative **110** were obtained from the organic extracts and from the water extracts of the soil fungus *T. flavus* [30, 34]. Another phthalide compound FKI-0076 B, vermistatin **111**, was obtained from *Talaromyces* sp. during the screening programme for synergist of azoles antifungal antibiotics [20]. **111** was also isolated from the extract of *T. flavus* IFM52668 [16], and from the culture broth *T. flavus* FKI-0076 which isolated from a soil sample [21]. Other two analogues penisimplicissin (**112**) and hydroxydihydrovermistatin (**113**) were isolated from the fungus *T. thailandiasis* [12].

Three new phthalide derivatives, talaromycolides A–C (**114**–**116**), and a known compound rubralide C (**117**), were isolated from the methanolic extracts of the green Chinese onion-derived fungus *T. pinophilus* AF-02. Talaromycolides A–C are rare phthalide derivatives with a novel linkage position between the phenyl and phthalide moieties, and exhibited significant antibacterial activity in response to some of the tested strains, *Bacillus subtilis*, *B. megaterium*, *Escherichia coli*, *Clostridium perfringens*, *Micrococcus tetragenus*, and no activity against the strain of MRSA (methicillin-resistant *Staphylococcus aureus*) [7].
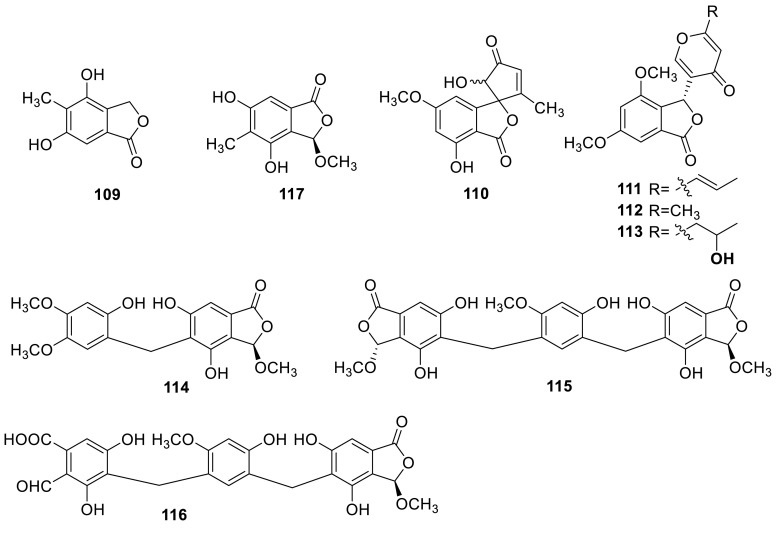


A six-membered ring lactone (**118**) was isolated from the water extracts of the soil fungus *T. flavus* [34]. Two lactones (**119** and **120**) were isolated from an endophytic fungus, a close relative of *Talaromyces* sp., found in association with *Cedrus deodara*. They displayed a range of cytotoxicities against human cancer cell lines (HCT-116, A-549, HEP-1, THP-1, and PC-3), and induced apoptosis in HL-60 cells, as evidenced by fluorescence and scanning electron microscopy studies [35]. In the course of screening for apoptosis inducers in ras dependent Ba/F3-V12 cells, a new active compound, rasfonin (**121**) was isolated from the fermented mycelium of *Talaromyces* sp. 3656-A1. The cytotoxic activity indicated that rasfonin induced cell death in Ba/F3-V12 cells in an IL-3-free medium containing Dex (2 × 10^−7^M) with an IC_50_ of 0.16 μg/mL and no cell death was observed in the presence of IL-3 at concentrations less than 1.25 μg/mL of rasfonin (IC_50_1.8 μg/mL) [36].

Wortmannilactones E–H (**122**–**125**), from the culture of the soil filamentous fungus *T. wortmannii*, showed inhibitory activities against cathepsin B with IC_50_ values of 4.3, 6.5, 13.0, and 6.0 μM, respectively [37]. In screening for NADH-fumarate reductase inhibitors led to the isolation of a new ukulactone analog, ukulactone C (**126**), as a major polyene compound produced by *Talaromyces* sp. FKI-6713. Ukulactone C possessed a potent inhibitory activity (IC_50_ 62 nM) against NADH-fumaratereductase of the roundworm *Ascaris suum* invitro [38].
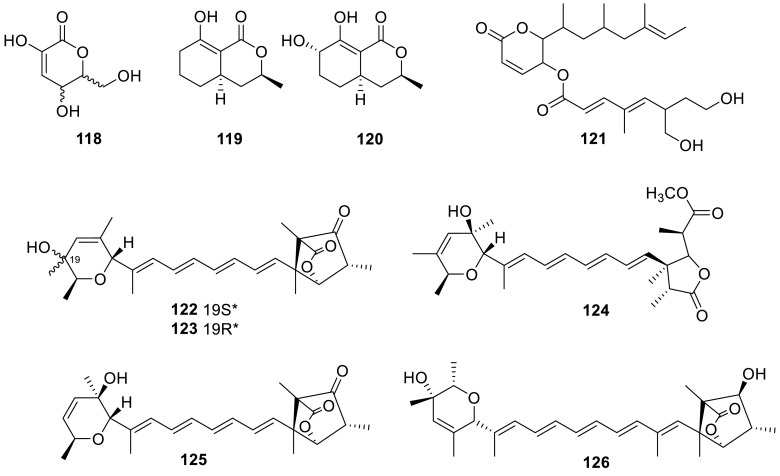


d-Glucono-1,4-lactone (**127**) was obtained from the organic extracts of the soil fungus *T. flavus* [30]. A new penicillic acid, coculnol (**128**) (five-membered ring lactone), was produced by a coculture of *Fusarium solani* FKI-6853 and *Talaromyces* sp. FKA-65. **128** showed an inhibitory effect (with IC_50_ value of 283 μg/mL) against A/PR/8/34 (H1N1) with weak cytotoxicity against MDCK cells (IC_50_ value of 781 μg/mL) [39]. Berkedienolactone (**129**) was isolated from the methanolic extracts of the green Chinese onion-derived fungus *T. pinophilus* AF-02 [7]. A new spiculisporic acid derivative, spiculisporic acid E (**130**), was isolated from the culture of the marine-sponge associated fungus *T. trachyspermus* (KUFA 0021) [40]. The ethoxylated of spiculisporic acid E (**131**) was isolated from the *T. panasenkoi* [41].
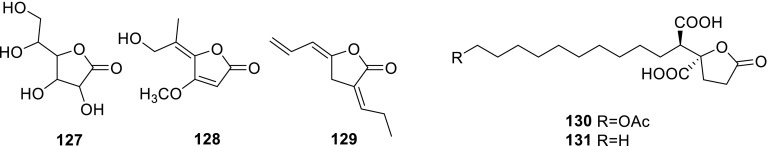


### Polyketides

Polyketides, pyrones, xanthones, are both a major focus of many research efforts and a rich source of novel metabolites of *Talaromyces.*

Hydroxymethylmaltol (**132**) was isolated from the water extracts of the soil fungus *T. flavus* [34]. Funicone (**133**) and a new funicone derivative, 9,14-epoxy-11-deoxyfunicone (**134**), were isolated from the strain *T. flaus* IFM52668. As the results of the antifungal assay showed that **133** had the characteristic inhibition against a human pathogenic filamentous fungus, *A. fumigates* (11-mm inhibition zone at 100 μg/disc), whereas **134** showed the weak antifungal activity against *A. niger* (10-mm inhibition zone at 200 μg/disc) [16]. Deoxyfunicone (**135**) and actofunicone (**136**) were obtained from the culture broth *T. flavus* FKI-0076 which isolated from a soil sample. **135** and **136** showed no effect on the growth of *Candida albicans* up to 300 μM, and a slight inhibition (35 %) was observed at that concentration for NG-012. But in the absence of the funicones, the IC_50_ value of miconazole against *C. albicans* was calculated to be 19 μM, however, in combination with the funicones (50 μM), the IC_50_ values were decreased to 1.6–3.7 μM, demonstrating that they reinforced the inhibition *C. albicans* activity of miconazole [20, 21].

Abenzopyrone derivate **137** was isolated from the organic extracts of the soil fungus *T. flavus* [30]. Benzopyrone derivatives **138** and **139** were isolated from a culture broth of a fungus, *Talaromyces* sp. **138** exhibited the weak anti-HBV activity with an IC_50_ value of 72.4 μM [42].
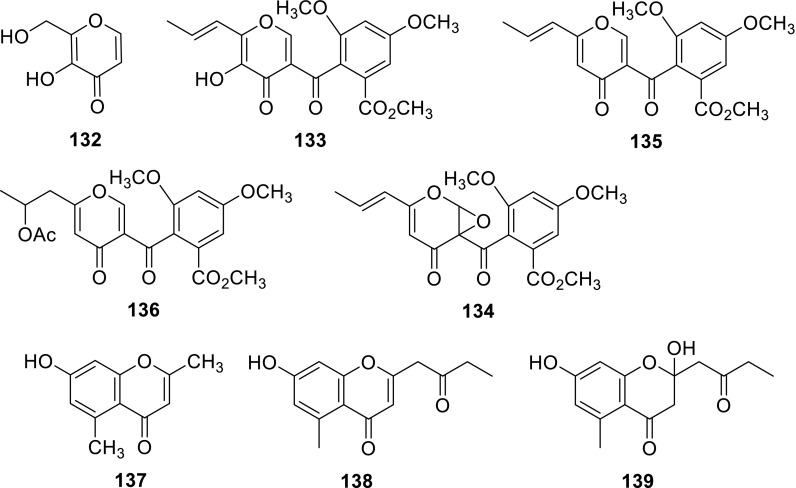


Two xanthones, norlichexanthone (**140**) and secalonic acid A (**141**), were obtained from the extract of the mangrove endophytic fungus *Talaromyces* sp. ZH-154 which was isolated from the stem bark of *Kandelia candel* (L.) Druce, Rhizophoraceae. **141** exhibited high activities against six selected strains. Moreover, in vitro cytotoxic activities indicated that **141** displayed very strong cytotoxicity against KB and KBv200 cell lines with IC_50_ values of 0.63 and 1.05 μg/mL, closed to those of the positive control (0.56 and 0.78 μg/mL). Whereas, the xanthone dimer **141** showed higher bioactivity than the xanthone monomer **140** [43].

A new isopentenylxanthenone, talaroxanthenone (**142**), was isolated from the culture broth and mycelia of a marine fungus *Talaromyces* sp. strain LF458. **142** displayed potent acetylcholinesterase inhibitory activities with IC_50_1.61 μM. Interestingly, phosphodiesterase PDE-4B2 was inhibited by compounds **142** (IC_50_ 7.25 μM) [26]. A new xanthone dimer talaroxanthone **143** was isolated from *Talaromyces* sp. which collected in the Amazonian rainforest from the medicinal plant *Duguetia stelechantha* [44].
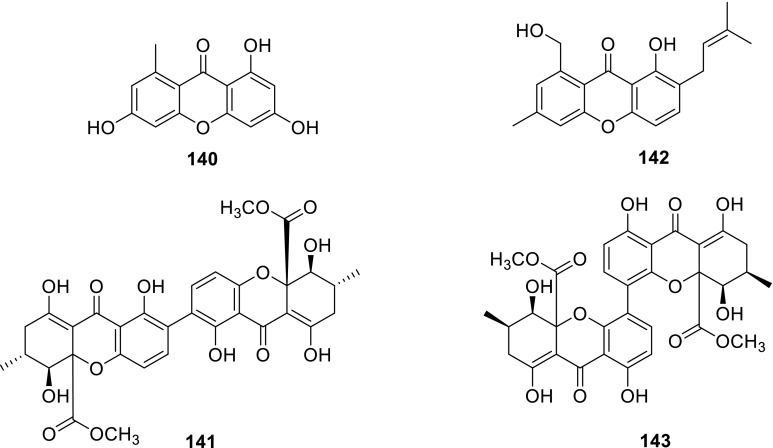


Two newpolyketides, 7-epiaustdiol (**144**) and 8-*O*-methylepiaustdiol (**145**), were obtained from the extract of the mangrove endophytic fungus *Talaromyces* sp. ZH-154 which was isolated from the stem bark of *Kandelia candel* (L.) Druce, Rhizophoraceae. **144** showed significant inhibitory activity to *Pseudomonas aeruginosa* with a MIC value of 6.25 μg/mL [43]. Two new polyketides, TL-1 and -2 (luteusins A and B) (**146** and **147**) with monoamine oxidase (MAO) inhibitory effect were isolated from an ascomycete *T. lutcus* [45]. Three new azaphilones, luteusins C, D, and E (**148**–**150**), together with **146** and **147**, were isolated from an Ascomycete, *T. luteus*. As regards MAO-inhibitory activity, the IC_50_ values of **146** and **147** were 6.6 and 11 μM, respectively [46].
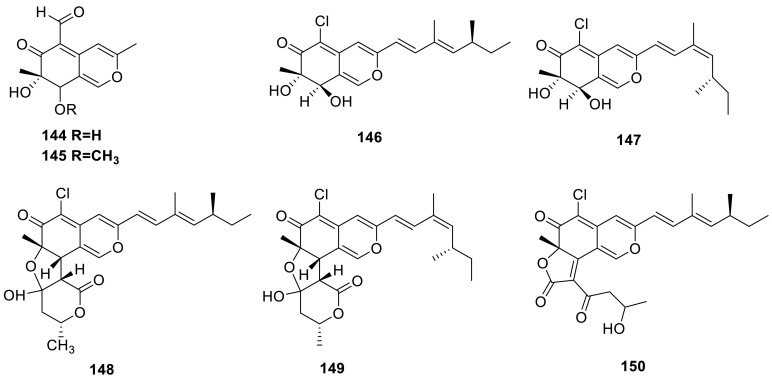


Kasanosins A (**151**) and B (**152**), novel azaphilones, were isolated from cultures of *Talaromyces* sp. derived from the seaweed. **151** and **152** selectively inhibited the activities of eukaryotic DNA polymerases β and λ (pols β and λ) in family X of pols, and **151** was a stronger inhibitor than **152**, and the IC_50_ values of **151** on rat pol β and human polλ were 27.3 and 35.0 μM, respectively. And the results also suggested that **151** and **152** could identify the inhibition between pols β, λ, and terminal deoxynucleotidyl transferase (TdT) in family X [47]. Kasanosin C (**153**) and entonaemin A (**154**) were isolated from the solid fermentation of *Talaromyces* sp. T1BF derived from the old bast tissue of *Taxus yunnanensis* [48]. A known polyketide (**155**) was isolated from the strain *T. wortmanii* [49]. Deacetylisowortmin (**156**) was isolated from the endophytic fungus *T. wortmannii* LGT-4 [50].
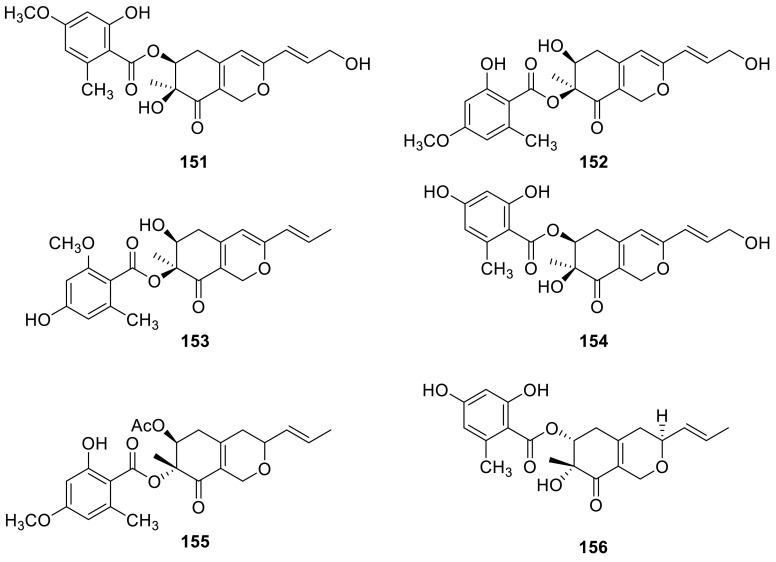


A new azaphilone derivative, monomethyl-(+)-mitorubrin (**157**), was isolated from the ascomata of *T. ardifaciens* derived from the paddy soil from Bhaktapur, Nepal [51]. Four new chlorinated azaphilones, helicusins A–D (**158**–**161**), were isolated from *T. helices*. **158**–**161** showed weak MAO-inhibitory effects [52]. Diazaphilonic acid (**162**) was obtained from *T. flavus* PF1195. **162** inhibited DNA amplification by polymerase chain reaction (PCR) with *Thermus thermophilus* DNA polymerase and the IC_50_ value was 2.6 μg/mL. **162** dose-dependently inhibited the telomerase activity of MT1 (human leukemia) and almost completely inhibited the activity at 50 μM. But **162** showed no antimicrobial activity [53].
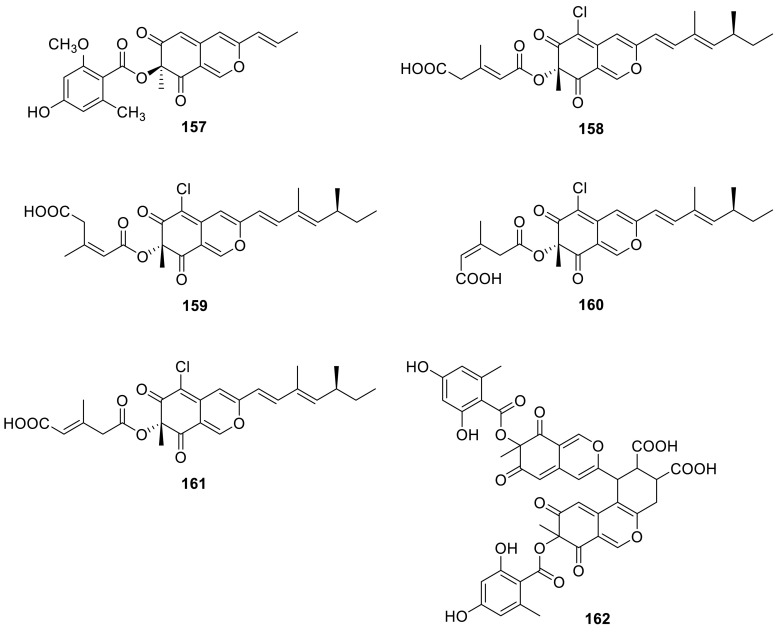


### Quinones

Three pigments, emodin (**163**), ω-hydroxyemodin (**164**), and emodic acid (**165**), were obtained from the strain *T. avellaneus* [54]. Emodin, erythroglaucin (**166**), and catenarin (**167**), were isolated from the strain *T. stipitatus* [55]. A new atropisomer, biemodin (**168**), as well as five known metabolites (**165** and **169**–**172**), was isolated from the strain *T. wortmannii*, an endophyte of *Aloe vera*. **169** and **171** exhibited considerable antibiotic activity against Gram positive pathogenic bacteria with MIC values ranging between 4 and 16 μg/mL. **168** also showed strong activity against Gram positive bacteria, especially against MRSA, but was less active compared to compounds **169** and **171** [49]. Emodin (**163**) and skyrin (**169**) were also isolated from the extract of the mangrove endophytic fungus *Talaromyces* sp. ZH-154 derived from *Kandelia candel* (L.) Druce [43]. Skyrin (**169**) was also isolated from the strain *T. wortmannii*, an endophyte of *Aloe vera* [56].
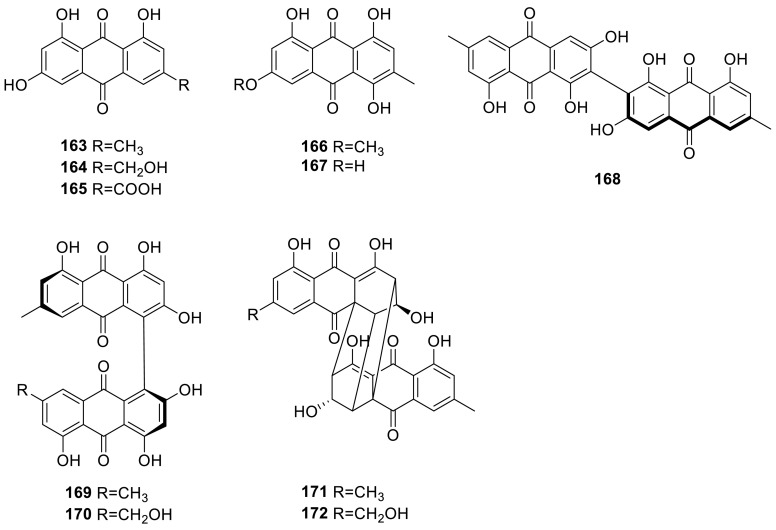


Two bisdihydroanthracenone atropodiastereomeric pairs, homodimeric flavomannin A (**173**) and flavomannin B (**174**), two new unsymmetrical dimers **175** and **176**, and two new mixed dihydroanthracenone/anthraquinone dimers **177** and **178**, were isolated from *T. wortmannii*, an endophyte of *Aloe vera*. The compounds exhibited antibacterial activity, including (multi) drug-resistant clinical isolates and compounds **173**–**178** were predominantly active against *Staphylococci*, with MIC values from 4 to 8 μg/mL. Reporter gene analyses indicated induction of the SOS response for some of the derivatives, suggesting interference with DNA structure or metabolism. But the compounds showed no cytotoxic activity, encouraging their further evaluation as potential starting points for antibacterial drug development [56].
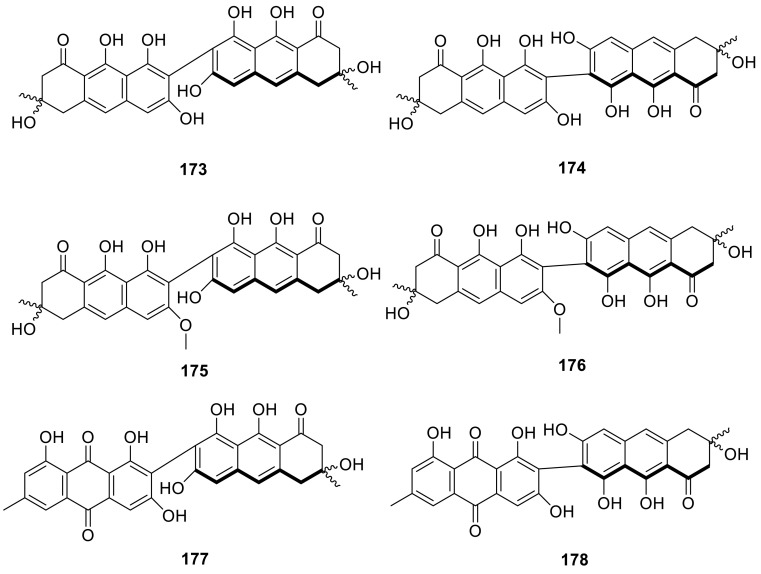


Two new tricyclic polyketides, vanitaracin A (**179**) and B (**180**), were isolated from a culture broth of a fungus, *Talaromyces* sp. **179** and **180** were evaluated for anti-HBV activity using HBV-susceptible HepG2-hNTCP-C4 cells and **179** exhibited the strong anti-HBV activity with an IC_50_ value of 10.5 μM [42]. Stemphyperylenol (**181**) was isolated from the extract of the mangrove endophytic fungus *Talaromyces* sp. ZH-154, and showed inhibitory activity against *Sarcina ventriculi* with a MIC value of3.12 μg/mL, lower than that of ampicillin (12.5 μg/mL) [43].
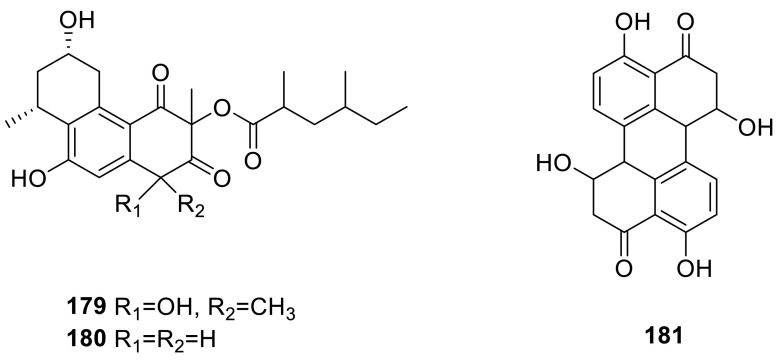


### Steroids and Terpenoids

A steroid **182** was isolated from the genus of *Talaromyces* sp. T1BF for the first time which isolated from an endophyte from *Taxus yunnanensis* by chromatography techniques [57]. A new natural product 3-acetyl ergosterol 5,8-endoperoxide (**183**) was isolated from the culture of the marine-sponge associated fungus *T. trachyspermus* (KUFA 0021) [40]. Secovironolide (**184**) was purified from the culture broth of *T. wortmanni* and is the first example of a furanosteroid scaffold bearing a five-membered B ring. Additional known viridian derivatives (**185**–**188**, **190**) were isolated, including the new epoxide containing compound, epoxyvirone (**189**). Isolates were tested and showed only weak MAO inhibitory activity [50].
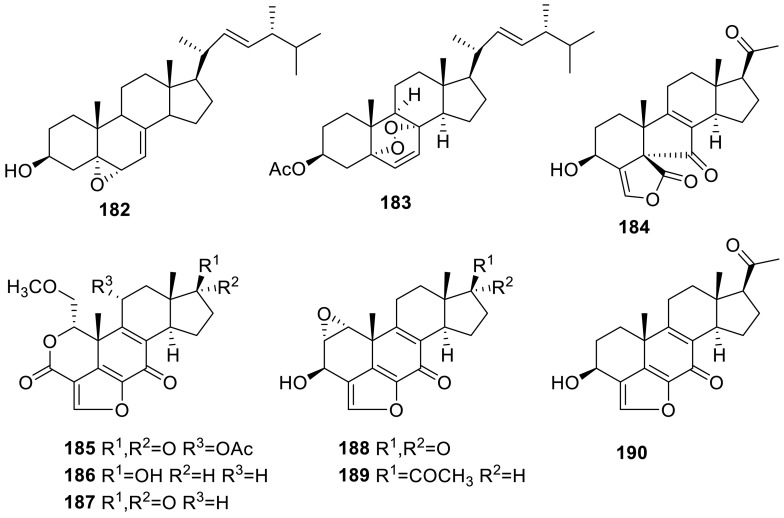


A new nardosinane-type sesquiterpene, talaflavuterpenoid A (**191**), was isolated from the wetland soil-derived fungus *T. flavus* BYD07-13. **191** was tested for the cytotoxic activity against five human tumor cell lines and the antimicrobial activity, however, **191 **showed no cytotoxic (IC_50_ > 40 μM) and antimicrobial activities (MIC > 1.0 mg/mL) [58]. Four new norsesquiterpene peroxides, named talaperoxides A–D (**192**–**195**), as well as a known analogue, steperoxide B (**196**), had been isolated from a mangrove endophytic fungus, *T. flavus*. Cytotoxic activities of **192**–**196** were evaluated in vitro against human cancer cell lines MCF-7, MDA-MB-435, HepG2, HeLa, and PC-3. **193** and **195** showed activity against the five human cancer cell lines with IC_50_ values between 0.70 and 2.78 μg/mL [59].
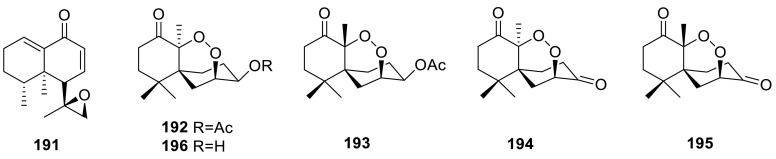


### Others

(−)-Epoformin(**197**) and (1S*,3R*,5R*)-3-methyl-2-oxa-bicyclo[3.3.1]nonan-7-one (**198**) were isolated from an endophytic fungus *Talaromyces* sp., found in association with *Cedrus deodara*. The sulforhodamine B cytotoxicity assay indicated that **197** was found to be the most active followed by compound **198** [35]. Four new spiroketaltalaromycins (**199**–**202**) had been isolated from the strain *T. stipitatus* [60]. A new metabolite, trachyspic acid (**203**) that inhibited heparanase, was isolated from the culture broth of *T. trachyspermus* SANK 12191. Its structure was determined from NMR spectral analyses and chemical reactions as a tricarboxylic acid derivative containing a spiroketal. The IC_50_ value of trachyspic acid against heparanase was 36 μM [61].
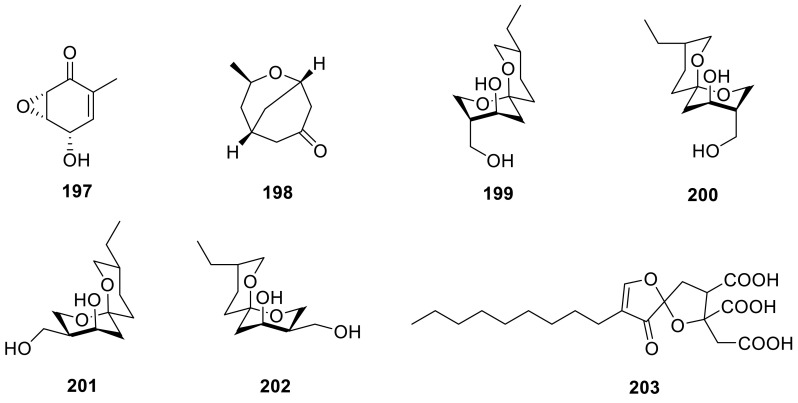


A novel benzene derivative (**204**) was isolated from a culture broth of a fungus, *Talaromyces* sp., and it was evaluated for anti-HBV activity using HBV-susceptible HepG2-hNTCP-C4 cells, but **204** exhibited the weak anti-HBV activity [42]. 5-Hydroxymethylfurfural (**205**) and two benzene derivatives **206** and **207** were isolated from the organic extracts of the soil fungus *T. flavus* [30]. **207** was also evaluated for its ability to inhibit HIV-1 integrase in coupled and strand-transfer assays and the data indicated that **207** with IC_50_ values of 19 μM in the coupled assay and 25 μM in the strand-transfer assay [62]. Two benzene derivatives **208** and **209** from the genus of *Talaromyces* sp. T1BF which isolated from an endophyte from *Taxus yunnanensis* by chromatography techniques [57].
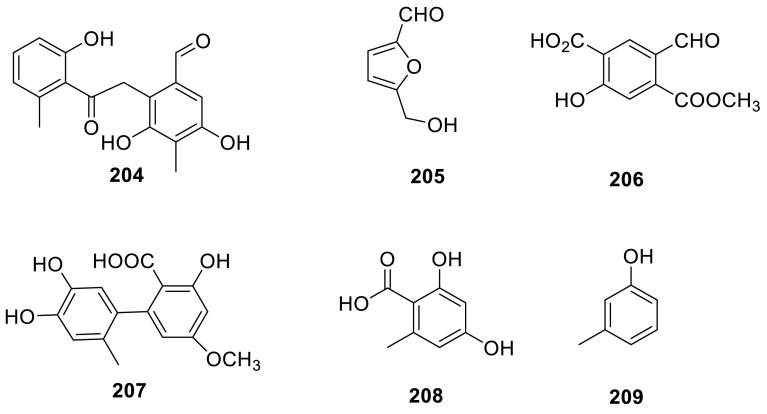


Three diphenyl ether derivatives including two new natural products, tenelates A (**210**) and B (**211**), together with the known compound, tenellic acid C (**212**), were isolated from the mangrove endophytic fungus *Talaromyces* sp. (SBE-14), from the South China Sea [63]. Three new derivatives of *p*-hydroxybenzoic acid (**213**–**215**) had been isolated from the culture filtrate of *T. derxii* [64].

A new long-chain dicarboxylic acid, 2-hydroxyradiclonic acid (**216**), and four known compounds, benzoic acid (**217**), (*Z*)-3-phenyl propenal (**218**), 2-formyl-3,5-dihydroxy-4-methylbenzoic acid (**219**), and radiclonic acid (**220**), were isolated from the methanolic extracts of the green Chinese onion-derived fungus *T. pinophilus* AF-02. **216** showed significant antibacterial activities against *E. coli* [7].

A new antibiotic, fosfonochlorin (**221**), was found in the culture filtrate of four strains of fungi freshly isolated from soil samples including *T. flavus*. The biological activity indicated that it was active against *Proteus mirabilis* and *P. vulgaris* and weakly active against *Salmonella enteritidis*, *Klebsiella pneumoniae* and *Providencia rettgeri*, and its synergistic effect with glucose-6-phosphate was observed on *Staphylococcus aureus* and *Escherichia coli* [65].
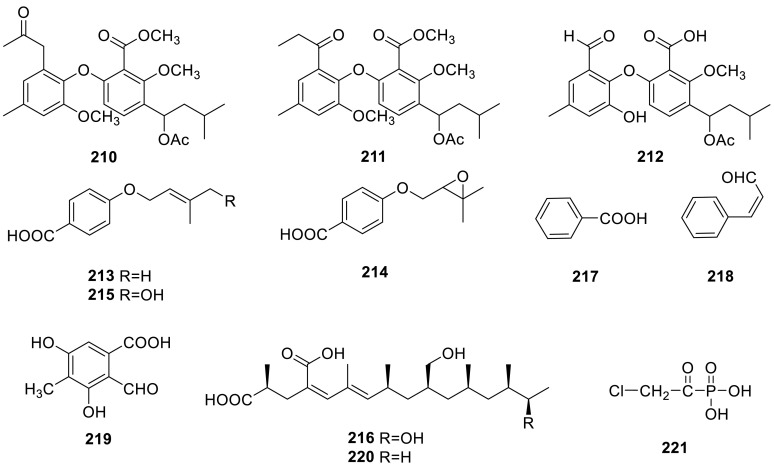


A new antifungal antibiotic, named talaron, had been isolated from the culture of *T. vermiculatus* (M-3224). Talaron is water-soluble acidic polysaccharide containing nitrogen and phosphorus, and its molecular weight was estimated to be 7000–8000. Talaron had strong fungicidal activity against filamentous dermatophytes and exhibited inhibitory activity against the spore germination of *Trichophyto asteroides* and showed cytotoxic effect at 1 mcg/mL on HeLa cells, and at 0.2 mcg/mL on mouse embryo fibroblast cells, but no antibacterial activity [66].

## Conclusions

The *Talaromyces* genus includes many species with a variety of uses, some of which are important in the food products and agriculture. Since, several anthraquinone metabolites from *T. avellaneus* were isolated in 1965 [54], lots of secondary metabolites described in this report were obtained from this genus fungi which from a soil sample, from the plant, or from a marine sponge. The 221 compounds, including 43 alkaloids and peptides, 88 esters, 31 polyketides, 19 quinones, 15 steroid and terpenoids, and 25 other structure compounds, described in this review were isolated from 28 species, which 19 species have been determined and 9 species were not given the specific names (Table 1). The secondary metabolite studies were mainly performed on the commonest species of the genus, *T. flavus* [3]. The stereochemistry of many compounds was determined via circular dichroism spectrum [7], Mosher’s analysis method [8], Marfey’s method [13], a single-crystal X-ray diffraction experiment using Cu Kα radiation [59], or quantum chemical calculation [6]. Those fungi were cultivated with varying media: potato dextrose, barley grains [10], rice [25], WSP30 [26], ISP_2_ broth [44], or other modified medium.

**Table 1:** The source of *Talaromyces* species

Species	Source	References
*Talaromyces* sp.		[6], [36], [20], [22], [42], [33]
	A soil sample	[14], [32], [38], [39]
	Marine sponge *Axinella verrucosa*	[26]
	Plants	
	*Cedrus deodara*	[35]
	*Duguetia stelechantha*	[44]
	*Kandelia candel*	[43], [63]
	*Taxus yunnanensis*	[48], [57]
	Sand	[42]
	Seaweed	[47]
*T. ardifaciens*	Paddy soil	[51]
*T. avellaneus*	A Soil sample	[54]
*T. bacillosporus*		[24]
	A soil sample	[25]
*T. convolutes*		[10]
*T. derxii*	A soil sample	[27], [29], [64]
*T. flavus*		[53], [62], [16]
	A soil sample	[21], [30], [34], [65]
	Wetland soil	[19], [31], [58]
	Leaves, *Sonneratia apetala*	[59]
*T. helices*		[52]
*T. luteus*		[45], [46]
*T. minioluteus*	A marine sponge	[11]
*T. panasenkoi*		[41]
*T. pinophilus*	A soil sample	[28]
	Green Chinese onion	[7]
*T. stipitatus*		[55], [60]
*T. tardifaciens*	Paddy soil	[51]
*T. thailandiasis*	A soil sample	[12]
*T. thermophilus*	Hot springs	[4], [5], [8], [9]
*T. trachyspermus*	Marine sponge *Clathria reianwardii*	[40]
	A soil sample	[61]
*T. vermiculatus*		[66]
*T. verruculosus*	Rhizosphere soil of *Stellera chamaejasme*	[15]
*T. wortmannii*	A soil sample	[17], [18], [37]
	Plants, *Aloe vera*	[13], [49], [56]
	Plants, *Tripterygium wilfordii*	[50]

In the early years of secondary metabolite of those genus species research was less emphasis on biological testing, but increasingly there has been a focus on the biological properties of these compounds. Inhibitory activity to tumour cells [17], bacteria [7], fungi [10], HBV [42], nematode [8], HIV-1-integrase [62], caspase-3 [11], mosquito larval [14], 5-lipoxygenase [27], and other activities were performed. Some of the isolated compounds have been used as pigments.

Studies on total synthesis and biotransformation of some of those compounds have been described. Structure–activity relationships have also been undertaken. Recently, there has been great interest in the study of biosynthesis genes based on secondary metabolites from the genus. However, systematic secondary metabolites–biosynthesis genes relationship might give insight into the molecular level, seem to be absent. This might be a promising direction in which work in the field of the secondary constituents from this genus fungi may proceed.
